# Endoscopic submucosal dissection of a gastric subepithelial lesion mimicking lipoma on endoscopic ultrasound: a rare case of gastric angiolipoma

**DOI:** 10.1055/a-2888-0628

**Published:** 2026-06-19

**Authors:** Yuanning Ye, Futao Wu, Siqi Yang, Lingling Guo, Jianqun Cai, Bo Fu, Aimin Li

**Affiliations:** 1 Department of GastroenterologyNanfang Hospital (Zengcheng Branch), Southern Medical UniversityGuangzhouChina; 2 Guangdong Provincial Key Laboratory of Gastroenterology, Department of GastroenterologyNanfang Hospital, Southern Medical UniversityGuangzhouGuangdongChina; 3 Department of PathologyNanfang Hospital (Zengcheng Branch), Southern Medical UniversityGuangzhouGuangdongChina

**Fig. 1:**
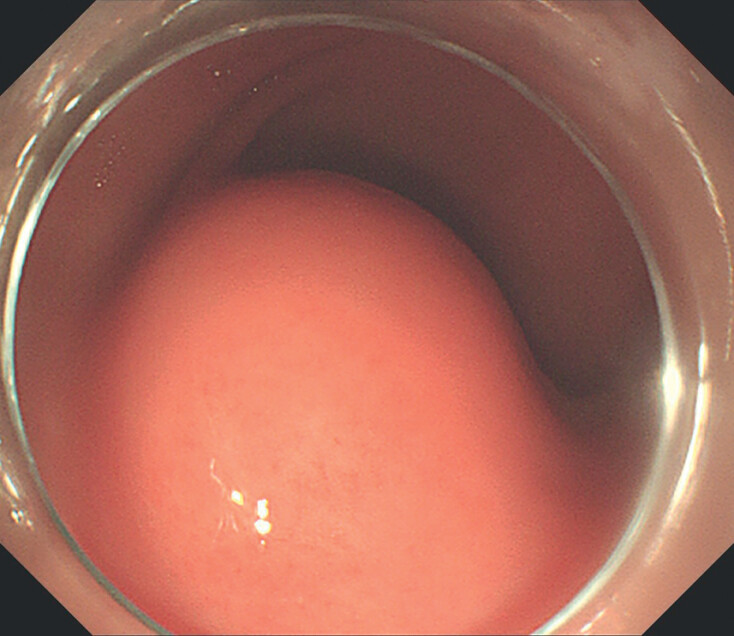
Endoscopic view of a subepithelial lesion in the gastric antrum under white-light imaging.

A 34-year-old man underwent upper gastrointestinal endoscopy during a routine health examination, which revealed a subepithelial lesion measuring approximately 30 mm in the gastric antrum ( Fig. 1 ). The patient was asymptomatic and laboratory findings were unremarkable.

**Fig. 2:**
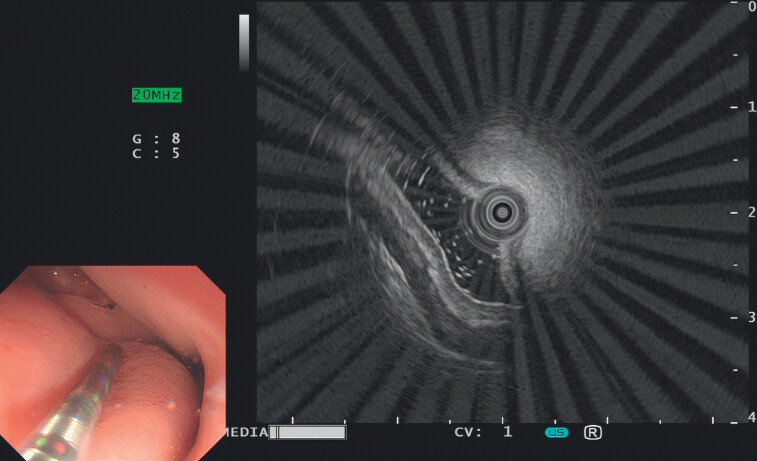
Endoscopic ultrasound demonstrating a well-defined homogeneous hyperechoic lesion arising from the submucosal layer.

Endoscopic ultrasound (EUS) using a 20-MHz miniature probe demonstrated a well-defined homogeneous hyperechoic lesion arising from the submucosal layer and suggestive of a gastric lipoma ( Fig. 2 ). However, the deep margin could not be completely visualized because of the limited penetration depth of the miniature probe, and diagnostic confidence remained insufficient.

Endoscopic submucosal dissection (ESD) was therefore performed for both diagnosis and treatment ( Video 1 ). A U-shaped mucosal incision was first created on the oral side of the lesion, followed by stepwise submucosal dissection from the oral to the anal side. During the procedure, prominent vascular structures were identified within the lesion, requiring careful coagulation to achieve hemostasis. En bloc resection was completed without perforation or delayed bleeding.

**Video 1** Endoscopic submucosal dissection of a gastric subepithelial lesion mimicking lipoma on endoscopic ultrasound: a rare case of gastric angiolipoma.


**Fig. 3:**
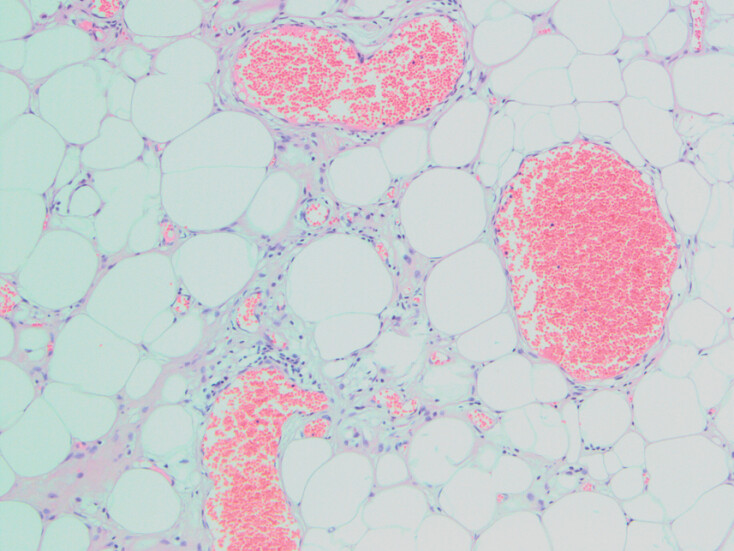
Histopathological examination revealing mature adipose tissue interspersed with proliferating blood vessels, consistent with gastric angiolipoma.

**Fig. 4:**
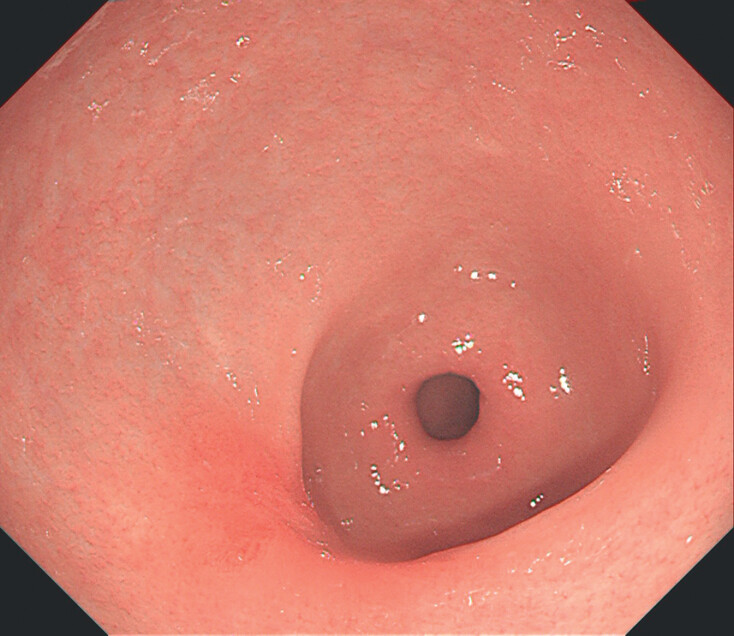
Follow-up endoscopy performed 3 months after endoscopic submucosal dissection demonstrating complete healing without recurrence.

The resected specimen measured approximately 50 mm in maximum diameter, including the surrounding mucosal margin. Histopathological examination revealed mature adipose tissue interspersed with proliferating blood vessels, confirming gastric angiolipoma ( Fig. 3 ). Follow-up endoscopy performed 3 months later demonstrated complete healing without recurrence ( Fig. 4 ).

Gastric angiolipoma may demonstrate variable EUS features depending on the relative proportion of adipose and vascular components. 1​ In the present case, incomplete visualization of the deep margin limited diagnostic confidence despite lipoma-like features on EUS. Previously reported gastric angiolipomas were predominantly managed surgically, particularly in symptomatic or bleeding cases. 2​ ESD enabled definitive diagnosis and complete minimally invasive resection in the present case, highlighting its potential role in selected gastric subepithelial lesions with uncertain diagnosis. 3​ In addition, the prominent vascular component identified during dissection underscores the importance of careful hemostatic management when resecting such lesions.

Endoscopy_UCTN_Code_CCL_1AF_2AD
